# Psychological Status of High School Students 1 Year After the COVID-19 Emergency

**DOI:** 10.3389/fpsyt.2021.729930

**Published:** 2021-10-14

**Authors:** Cong Zhou, Rongqin Li, Mingchuan Yang, Shanshan Duan, Chuanming Yang

**Affiliations:** ^1^School of Mental Health, Jining Medical University, Jining, China; ^2^Department of Psychology, Affiliated Hospital of Jining Medical University, Jining, China; ^3^Shandong Daizhuang Hospital (Jining Psychiatric Hospital), Jining, China; ^4^Jining Yucai High School, Jining, China; ^5^University of Mississippi, Oxford, MS, United States; ^6^The First High School of Jiaxiang, Jiaxing, China

**Keywords:** COVID-19, high school students, psychological status, mental health, depression, anxiety

## Abstract

**Background:** With the control of the epidemic, adolescents' mental outlook might have improved. However, little evidence existed with regard to the psychological status of adolescents in post-COVID-19 era. This present study aimed to explore the psychological status of high school students after the epidemic getting eased.

**Methods:** A web-based cross-sectional survey was used to obtain data from three high schools, including the demographic information, the Patient Health Questionnaire-9 (PHQ-9), the Generalized Anxiety Disorder-7 (GAD-7), the Self-Rating Scale of Sleep (SRSS), and self-designed general recent-status questionnaire. Correlation analysis was performed to explore potential associations between the depression symptoms, anxiety symptoms, and sleep status. The PHQ-9 and GAD-7 differences between nowadays data and the data enrolled 12 months before were also compared.

**Result:** A total of 1,108 qualified questionnaires were obtained. The prevalence of depressive and anxious symptoms was 27.5 and 21.3%, respectively, from mild to severe in all students, while 11.8% of these high students got sleep disturbances. Both the rate and the severity of depression, anxiety and sleep problems of female students were higher than male students. Grade three students suffered higher prevalence and severer mental disturbances than the other two grades. There were significant correlations between the depression symptoms, anxiety symptoms, and sleep status. The psychological status has been improved in nowadays high school students compared with the sample enrolled 12 months before.

**Conclusion:** As a supplement to our former study, this present research provided a perspective on the psychological status of high school students 1 year after the COVID-19 pandemic being well controlled. We should pay attention to the psychological status of high school students, and should also notice the progresses made by this special group after the epidemic.

## Introduction

The coronavirus disease 2019 (COVID-19) pandemic has swiped around the world for nearly one and a half years, resulting in over 150 million confirmed cases together with 3,159,547 deaths globally as of April 30th, 2021, according to the data from the World Health Organization (https://covid19.who.int/). Besides damages to the respiratory system caused by the severe acute respiratory syndrome coronavirus 2 (SARS-Cov-2), emerging evidence indicated that the virus could also invade the central nervous system (CNS), leaving behind some neurological and psychiatric symptoms (1–3). In order to stop the spread of the epidemic, a range of public health interventions have been applied including containment, quarantine, community control, and school closures, which has achieved remarkable results (4). However, the potential effects of social isolation on mental health should not be ignored (5). Urgent quarantine and isolation measures may have a negative psychological and social impact especially on the most vulnerable people, such as front-line medical workers, the elderly, and children and adolescents (6–9).

Generally speaking, children and adolescents are at a relatively high risk of being depressive and anxious even under the circumstance of no epidemic situation (10, 11). Isolation and distance learning during the epidemic might aggravate potential mental problems. Adolescents were more likely to report moderate to severe symptoms of depression, anxiety, posttraumatic stress disorder, suicidal ideation or behavior, and sleep problems compared to adults since the COVID-19 pandemic (12). Long-time social isolation and dissatisfaction with online learning may worsen emotional problems (13, 14). Some literatures reported the rates of depression and anxiety were between 12.33–57 and 6.26–36.7%, respectively, in Chinese children and adolescents during the COVID-19 pandemic (14–18). Above inconsistencies of different studies might be caused by the heterogeneity of study samples, various time nodes of data collection, and the diversity of measurement scales. Nevertheless, the findings were still astonishing and thought-provoking.

High school students in China are a special group bearing the huge pressure of Chinese National College Entrance Exam, and is thought to have increased risk of psychological distress than students in primary school (18). At the early May last year, we conducted a cross-sectional survey (19) on high school students in Shandong Province, China. We found that the prevalence of depression, anxiety, and the combination of depressive and anxiety symptoms of this special group was as high as 52.4, 31.4, and 26.8%, respectively, during the COVID-19 pandemic outbreak. At that time, the implementation of quarantine in China has lasted for over 3 months, and the epidemic in Shandong Province had tended to be moderated. Grade three students already went back to school normally for 2 weeks, while grade one and grade two students were still in quarantine and studying online. This might account for the reasons that grade three students who were the closest to the college entrance examination suffered less psychiatric symptoms instead. Besides, female students exhibited a higher rate and severity of depression and anxiety than male, which is consistent with some previous studies (16, 20) and the theory that females are more susceptible to stress exposure (21, 22). This study reminded us that sufficient attentions should be paid to the psychological status of high school students.

With the alleviation of the epidemic, the release of isolation, and the resumption of classes, adolescents' mental outlook might have improved. However, little evidence existed with regard to the psychological status of adolescents in post-COVID-19 era. This present study aimed to explore the psychological status of high school students in Shandong Province including depression symptoms, anxiety symptoms, and insomnia symptoms 1 year after they returned to school and resumed classes. We also promoted a self-designed general recent-status questionnaire to investigate the self-evaluation of their study effect and life attitude, as well as knowledge on COVID-19 pandemic. In addition, we further investigated the depression and anxiety symptoms differences between nowadays high school students and the sample which enrolled 12 months before. This will form a sequential study which can provide a comprehensive perspective of the psychological status of high school students both during the pandemic outbreak and after the epidemic getting eased. We speculated that as the epidemic has been under control and with the students returning to school normally, the psychological status of high school students might be improved.

## Materials and Methods

### Participants

The studies involving human participants were reviewed and approved by the Ethics Committee of the Shandong Daizhuang Hospital (Jining Psychiatric Hospital). Written informed consent was received online before the respondents began the questionnaire. This study was in accordance with the American Association for Public Opinion Research (AAPOR) reporting guideline. This was an anonymous survey, and confidentiality of data was ensured.

The cross-sectional online survey was conducted from May 6th to May 14th, 2021 in three high schools in Shandong Province, consistent with our previous study (19). The same investigation tool known as “Questionnaire Star” (https://www.wjx.cn/) was used to send questionnaire and collect data from the participants. Finally, 1,108 students submitted the questionnaire, and all questionnaires were qualified.

Last year's data was collected from high school students in Shandong Province from May 1st to May 7th, 2020, mainly investigating the students' depression and anxiety status at that time (21).

Both studies inclusion criterion was high school students who voluntarily participate in the mental health assessments. Exclusion criteria were (1) present or previous history of other psychiatric or neurological illness or serious physical disease and (2) not in Shandong Province.

### Measurement Tools

The demographic and neuropsychological data from the respondents were obtained by using the questionnaire. In addition to the general demographic information (grade, age, gender, current residence, and history of close contact to SARS-CoV-2), the Patient Health Questionnaire 9-item (PHQ-9) and the Generalized Anxiety Disorder scale (GAD-7) were also applied to obtain the psychological status. The total score indicates different levels of depressive or anxious symptoms: minimal/no depression (0–4), mild (5–9), moderate (10–14), or severe (≥15). We also evaluated the sleep status by using the Self-Rating Scale of Sleep (SRSS). The SRSS is designed to assess the sleep quality in different populations. There are 10 items in total with each item having a 5-point scale (1–5). The higher the score, the worse the sleep problems (23). We defined cases with sleep problems as a total score of SRSS ≥ 23 (24).

We also investigated the general recent-status of these students including their study effects, interpersonal relationship, and life attitudes for a preliminary view. Details could be found in the Supplementary Material.

### Statistical Analysis

The statistical analyses were performed using IBM SPSS Statistics (version 21.0; IBM, Armonk, NY, USA). Same with our previous research, the categorical variables were expressed as the frequency (%), and the continuous variables were presented as mean ± SD. Differences in PHQ-9, GAD-7, and SRSS scores between male students and female students were evaluated using the Mann-Whitney *U* test. Differences in scores among three grades were assessed using the Kruskal-Wallis test. We also conducted spearman's correlation analysis to explore the association between depression level, anxiety level, as well as recent-status survey scores. A two-tailed *P* < 0.05 was considered statistically significant.

Besides statistical analysis on present data, we further investigated age, gender, PHQ-9, and GAD-7 differences between nowadays data and the data which we collected at last year in the whole group. The Mann-Whitney *U* test and chi-square test were used to achieve above procedures.

## Results

### Demographic Characteristics

A total of 1,108 qualified questionnaires were obtained. The average age of the respondents was 16.39 ± 0.80 (years); 50.9% of them were female. The respondents all lived in Shandong Province and 88.8% of whom lived in the city. Four students got a history of close contact to SARS-CoV-2. Table 1 shows the detailed demographic characteristics of the participants.

**Table 1:** Demographic characteristics of the sample.

**Variables**	**All**	**Grade one**	**Grade two**	**Grade three**
Total number	1,108	720	276	112
**Gender**				
Male, *n* (%)	544 (49.1)	387 (53.8)	109 (39.5)	48 (42.9)
Female, *n* (%)	564 (50.9)	333 (46.2)	167 (60.5)	64 (57.1)
Age (years)	16.39 ± 0.80	16.01 ± 0.56	16.78 ± 0.49	17.86 ± 0.50
**Current residence**				
City, *n* (%)	984 (88.8)	629 (87.4)	253 (91.7)	102 (91.1)
Rural areas, *n* (%)	124 (11.2)	91 (12.6)	23 (8.3)	10 (8.9)
**History of close contact to SARS-CoV-2**				
Yes, *n* (%)	4 (0.4)	3 (0.4)	1 (0.4)	0 (0)
No, *n* (%)	1104 (99.6)	717 (99.6)	275 (99.6)	112 (100)

### Psychological Status

The prevalence of depressive and anxious symptoms was 27.5 and 21.3%, respectively, from mild to severe in all students, while 11.8% of these high students got sleep disturbances. Both the rate and the severity of depression, anxiety, and sleep problems of female students were higher than male students. Grade three students suffered higher prevalence and severer mental disturbances than the other two grades. The detailed results were exhibited in Table 2.

**Table 2:** The rate of different psychological symptoms in high school students assessed by PHQ-9, GAD-7, and SRSS.

**Variables**	**All**	**Gender**		***P***	**Grade**			***P***
	**(*n* = 1,108)**	**Male**	**Female**		**Grade one**	**Grade two**	**Grade three**	
		**(*n* = 544)**	**(*n* = 564)**		**(*n* = 720)**	**(*n* = 276)**	**(*n* = 112)**	
**PHQ-9, depression symptoms**								
Mean score	2.91 ± 3.59	2.51 ± 3.60	3.29 ± 3.54	<0.0001	2.61 ± 3.38	2.51 ± 3.88	3.33 ± 3.93	0.0006^**^
Minimal/ No depression	803 (72.5)	422 (77.6)	381 (67.6)		546 (75.8)	181 (65.6)	76 (67.9)	
Mild	252 (22.7)	99 (18.2)	153 (27.1)		147 (20.4)	75 (27.2)	30 (26.8)	
Moderate	41 (3.7)	16 (2.9)	25 (4.4)		21 (2.9)	16 (5.8)	4 (3.6)	
Severe	12 (1.1)	7 (1.3)	5 (0.9)		6 (0.8)	4 (1.4)	2 (1.8)	
Mild to severe	305 (27.5)	122 (22.4)	183 (32.4)		174 (20.2)	95 (34.4)	36 (32.1)	
**GAD-7**,								
**anxiety symptoms**								
Mean score	2.15 ± 3.07	1.81 ± 3.04	2.48 ± 3.07	<0.0001	1.88 ± 2.88	2.58 ± 3.32	2.88 ± 3.37	<0.0001
Minimal/No depression	872 (78.7)	451 (82.9)	421 (74.6)		590 (81.9)	201 (72.8)	81 (72.3)	
Mild	210 (19.0)	81 (14.9)	129 (22.9)		118 (16.4)	64 (23.2)	28 (25.0)	
Moderate	19 (1.7)	7 (1.3)	12 (2.1)		8 (1.1)	9 (3.3)	2 (1.8)	
Severe	7 (0.6)	5 (0.9)	2 (0.4)		4 (0.6)	2 (0.7)	1 (0.9)	
Mild to severe	236 (21.3)	93 (17.1)	143 (25.4)		130 (18.1)	75 (27.2)	31 (27.7)	
**SRSS**								
Mean score	17.20 ± 4.53	16.72 ± 4.59	17.67 ± 4.43	<0.0001	16.98 ± 4.33	17.41 ± 4.57	18.10 ± 5.54	0.009^*^
No sleep problems	977 (88.2)	487 (89.5)	490 (86.9)		636 (88.3)	245 (88.8)	96 (85.7)	
With sleep problems	131 (11.8)	57 (10.3)	74 (13.1)		84 (11.7)	31 (11.2)	16 (14.3)	

*PHQ-9, Patient Health Questionnaire 9-item; GAD-7, Generalized Anxiety Disorder scale; SRSS, Self-Rating Scale of Sleep*.

* *P <0.01*.

** *P <0.001*.

Among the depressive symptoms revealed by PHQ-9, the most common one is “Feeling tired or having little energy” (41.9%), while the least common one is “Thoughts that you would be better off dead, or of hurting yourself in some way” (10.6%). Among the anxious symptoms revealed by GAD-7, the most common one is “Feeling nervous, anxious or on edge” (37.0%). The least common one is “Feeling afraid as if something awful might happen” (17.3%). See Supplementary Tables 1, 2. The rate of comorbid depressive and anxiety symptoms among the students was 17.7% from mild to severe. Female students got a higher prevalence than male, while grade two students got a higher rate than the other two grades (see Table 3).

**Table 3:** The rate of comorbid depression and anxiety symptoms in high school students.

**Variables**	**All**	**Male**	**Female**	**Grade one**	**Grade two**	**Grade three**
	**(*n* = 1,108)**	**(*n* = 544)**	**(*n* = 564)**	**(*n* = 720)**	**(*n* = 276)**	**(*n* = 112)**
Comorbid depression and anxiety symptoms (mild to severe)	196 (17.7)	76 (14.0)	120 (21.3)	109 (15.1)	64 (23.2)	23 (20.5)

### General Recent-Status

Most of the students (71.3%) considered that the efficiency of studying at school was better than online-study during home quarantine (Question 1). Fifty-nine percent of them thought that the interactions between students and teachers during school time became more active than online-study during quarantine (Question 4). Nearly 2/3 students had a good relationship with their family and classmates since the pandemic being under good control (Question 8 and 9). More than half of the students got a more positive life attitude after the pandemic (Question 10). Most of the students (68.9%) spent little time focusing on COVID-19 related information (Question 11), and few students (1.9%) often or always felt scared or anxious or confused about COVID-19 related news (Question 12). See Supplementary Table 3 for more details.

### Correlations Between Depression Symptoms, Anxiety Symptoms, and Sleep Status

The PHQ-9 score had a strong positive correlation with the GAD-7 score (*r* = 0.814, *P* < 0.0001) and a moderate positive correlation with the SRSS (*r* = 0.547, *P* < 0.0001). The GAD-7 score had a moderate positive correlation with the SRSS (*r* = 0.573, *P* < 0.0001). The detailed results were exhibited in Table 4.

**Table 4:** Correlations between PHQ-9, GAD-7, and SRSS.

	**PHQ-9**		**GAD-7**		**SRSS**	
	***r***	***P***	***r***	***P***	***r***	***P***
PHQ-9	1	-	0.814	<0.0001	0.547	<0.0001
GAD-7	0.814	<0.0001	1	-	0.573	<0.0001
SRSS	0.547	<0.0001	0.573	<0.0001	1	-

*PHQ-9, Patient Health Questionnaire 9-item; GAD-7, Generalized Anxiety Disorder scale; SRSS, Self-Rating Scale of Sleep*.

### Depression and Anxiety Symptoms Differences Between Nowadays Students and the Students in Last Year

**Figure 1:**
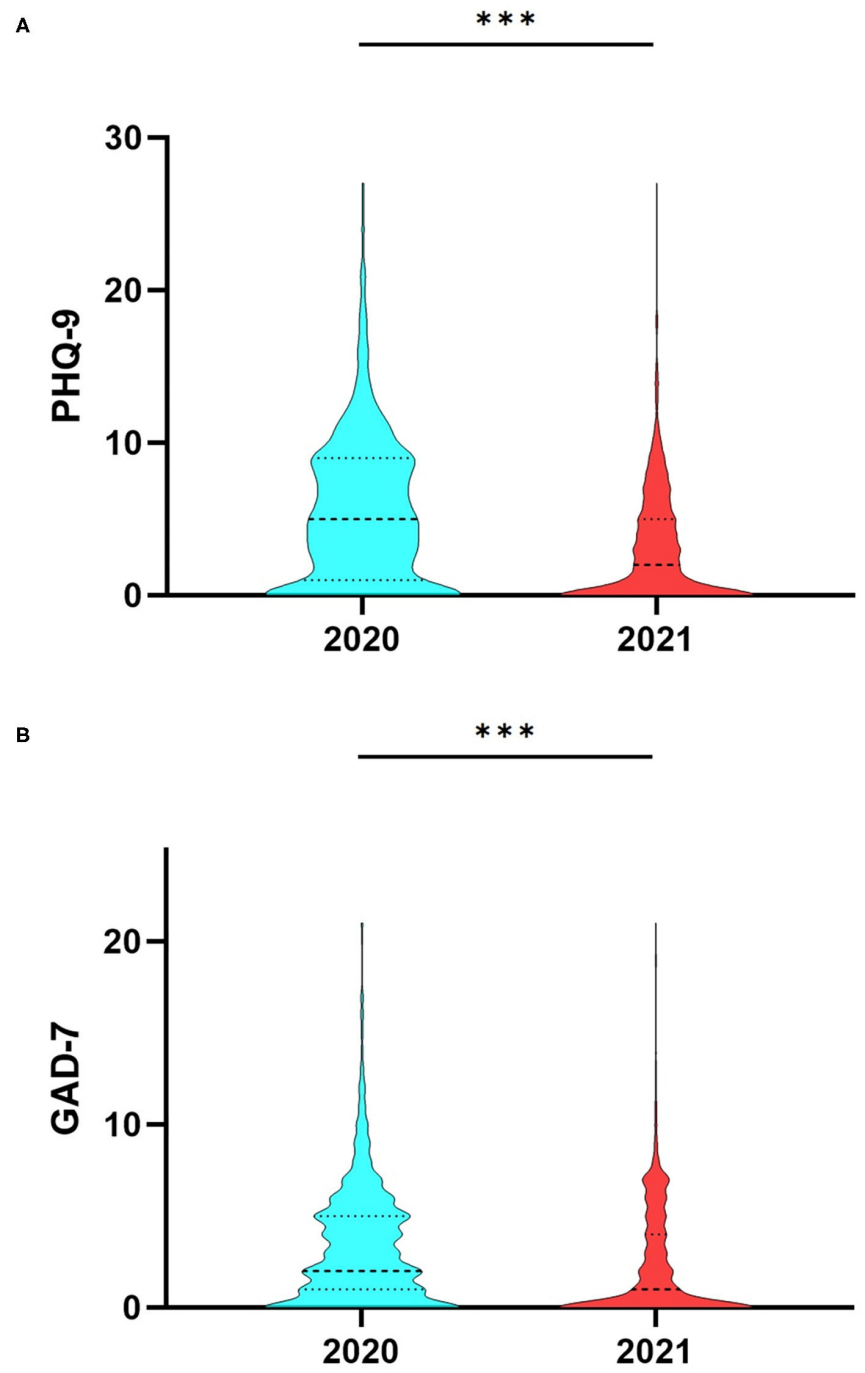
**(A)** PHQ-9 and **(B)** GAD-7 differences between the students in 2020 and the students in 2021. PHQ-9, Patient Health Questionnaire 9-item; GAD-7, Generalized Anxiety Disorder scale. ***P < 0.0001.

Compared with last year's data, both the PHQ-9 (5.49 ± 4.81 vs. 2.91 ± 3.59, *P* < 0.0001) and the GAD-7 (3.25 ± 3.25 vs. 2.15 ± 3.07, *P* < 0.0001) were significantly decreased in nowadays students (see Table 5 and Figure 1).

**Table 5:** The demographic characteristics and depression and anxiety symptoms differences between high school students in 2020 and 2021.

**Variables**	**Students in 2020**	**Students in 2021**	***Z/*χ2**	***P***
	**(*n* = 1,018)**	**(*n* = 1,108)**		
Age (years)	16.61 ± 1.06	16.39 ± 0.80	5.546	<0.0001
Gender (male/female)	473/545	544/564	1.475	0.225
PHQ-9 mean score	5.49 ± 4.81	2.91 ± 3.59	−13.887	<0.0001
GAD-7 mean score	3.25 ± 3.25	2.15 ± 3.07	−10.630	<0.0001

*PHQ-9, Patient Health Questionnaire 9-item; GAD-7, Generalized Anxiety Disorder scale*.

## Discussion

One year after the COVID-19 pandemic being well controlled, high school students in Shandong Province showed that the prevalence of depressive and anxious symptoms was 27.5 and 21.3%, respectively, from mild to severe, while 11.8% of these high students got sleep disturbances. Female students and grade three students suffered more mental distresses than male and other two grades. Most of the students got a high evaluation of their general recent-status, such as feeling better studying at school than online-study during home quarantine and having a more positive life attitude than pandemic time. There were significant associations between their psychological status and general recent-status.

Social isolation and quarantine measures could result in the emergence of psychological disturbances, and worsen existing mental problems among children and adolescents (25). However, with the release of isolation and the resumption of classes, psychological status of high school students might become reversed. Our present research found that compared with last year's data, both the depression and anxiety symptoms were significantly relieved in nowadays students. According to our previous research, during the outbreak of the COVID-19 epidemic, only 47.6% of the high school students in Shandong Province exhibited no depressive symptoms and only 68.9% of them reported no anxious symptoms (19). These rates have increased to 72.5 and 78.8%, respectively, based on our present statistics, close to pre-COVID-19 times (11). The most common depression manifestation in nowadays students is “Feeling tired or having little energy,” same with last year. However, the most common anxiety manifestation has changed to “Feeling nervous, anxious or on edge” instead of last year's “Being so restless that it is hard to sit still”. Besides, incidence of sleep problems is 11.8% in today's high students, and most of them felt not getting enough sleep and could only sleep <7 h a day. In consideration of the characteristics of this special group, it seems that the major sources of depression and anxiety has changed from the COVID-19 to normal pressure of the Chinese National College Entrance Exam or daily study assignments. The findings that most of the students spent little time focusing on COVID-19 related information and most of them did not feel scared or anxious or confused about COVID-19 related news supported our conjectures. It is noteworthy that the rates of depression, anxiety, and sleep problems were consistently lower with respect to last year, but was still at a relatively high level. Mental health of high school students should still not be neglected.

When talking about the vulnerable groups of this population, it seemed that girls were still more likely to be depressed and anxious than boys. Both the depression and anxiety rate and the symptom severity of female students were higher than male students just like last year. Besides, girls suffered more sleep disturbances, consistent with previous study (21, 22). When it comes to the grade, grade three students became the most susceptible grade to mental distress than the other two grades. By the time we conducted our investigation, it is only 1 month away from the Chinese National College Entrance Exam. Grade three students were bearing more pressure, which might be the cause of more serious psychological problems.

Chronic social isolation and loneliness are associated with lower physical and mental health (26). Normal learning style and healthy social activities are significant to stable emotions and good psychological status. In the present study, there were strong correlations between the psychological status and general recent-status of these students. Most of them considered a better efficiency of studying at school than online-study during home quarantine, and 59% of them thought that the interactions between students and teachers during school time became more active than online-study. Satisfactory study effect and learning atmosphere might bring about the improvement of mental outlook. Positive social interactions in and of themselves may be basic human needs analogous to other basic needs like food consumption or sleep (27). With the release of isolation and the restoration of social contact, nearly 2/3 students had a good relationship with their family and classmates. Despite all the sorrows and damages to the people, the pandemic offered an opportunity for young people to develop and hone their resilience and adaptability, and appreciate the value of life (28). We were gratified that after this special experience, more than half of the students got a more positive life attitude.

There were some limitations that should be addressed. Firstly, the information about the students' parental educational level, socioeconomic status, parental work, and the teachers' psychological status were still not collected. The study would be much more valuable if above contents could be explored. Secondly, the general recent-status was acquired by a self-designed questionnaire, and the psychometric characteristics of the questionnaire were not analyzed, which might have a certain result deviation. Last but not least, though the comparisons between nowadays data and last year's data were performed, strictly speaking, this study was still not a follow-up study and the investigation was not longitudinal, as the students in each grade had been entered to a higher grade, and the grade three students in last year had been graduated from high school for nearly 1 year at present. So, it is not able to track everyone's psychological status to provide targeted supports and assistants.

## Conclusion

As an important supplement to our former study, this present research provided a perspective on the psychological status of high school students 1 year after the COVID-19 pandemic being well-controlled. Compared with last year's data, both the prevalence and the severity of mental symptoms were decreased in nowadays high school students. Most of the students showed a more positive attitude of their general recent-status than pandemic time. The psychological status of high school students should attract sufficient attentions. Meanwhile, we should also notice the progresses and improvements made by this special group.

## Data Availability Statement

The original contributions presented in the study are included in the article/Supplementary Material, further inquiries can be directed to the corresponding authors.

## Ethics Statement

The studies involving human participants were reviewed and approved by the Ethics Committee of Shandong Daizhuang Hospital. Written informed consent to participate in this study was provided by the participants' legal guardian/next of kin.

## Author Contributions

CZ designed the study, wrote the initial manuscript, and revised the manuscript. RL, MY, and CY collected the data and undertook the statistical analysis. RL and SD interpreted the data and modified the paper. All authors contributed to and have approved the final manuscript.

## Funding

This study was supported by the Supporting Fund for Teachers' Research of Jining Medical University (600903001).

## Conflict of Interest

The authors declare that the research was conducted in the absence of any commercial or financial relationships that could be construed as a potential conflict of interest.

## Publisher's Note

All claims expressed in this article are solely those of the authors and do not necessarily represent those of their affiliated organizations, or those of the publisher, the editors and the reviewers. Any product that may be evaluated in this article, or claim that may be made by its manufacturer, is not guaranteed or endorsed by the publisher.
