# Supplementary material for: Psychological Status of High School Students 1 Year After the COVID-19 Emergency
Source: Front Psychiatry. 2021 Oct 14;12:729930. doi: 10.3389/fpsyt.2021.729930 (PMC8551869; doi:10.3389/fpsyt.2021.729930)
Supplement: Supplementary file 1 [file Data_Sheet_1.doc]

**Supplementary Material**

**General Recent-Status**

**Questionnaire Design**

The self-designed general recent-status questionnaire consisted of questions about study effect and life attitude, and was almost the same as the one we used last year, unless several questions about feelings during home quarantine have been changed and two questions about knowledge on COVID-19 pandemic were added. The final 12 questions were: 1). What do you think of the efficiency of studying at school compared with the online-study during home quarantine? Options: ①Higher; ②Almost the same; ③Lower. 2). How long do you study at school every day? Options: ①More than 10 hours; ②8-10 hours; ③6-8 hours; ④Less than 6 hours. 3). Could you finish your homework on time? Options: ①Always; ②Often; ③Only sometimes; ④Never. 4). How is the interaction between you and your teachers during school study compared with online-study during quarantine? Options: ①More interactive than before; ②Almost the same; ③Less interactive than before; ④Little interaction. 5). How much could you master from the online-study? Options: ①More than 90%; ②65-90%; ③40-65%; ④Less than 40%. 6). Are you tired of studying at school? Options: ①Never; ②Only sometimes; ③Often; ④Always. 7). Compared with studying at school in a normal way, do you like remote online-study better? Options: ①Never; ②Only sometimes; ③Often; ④Always. 8). How is your relationship with your family since the pandemic being under good control? Options: ①Always harmonious; ②Not bad; ③Not quite good; ④Poor. 9). How is your relationship with your classmates since returning to school? Options: ①Always harmonious; ②Not bad; ③Not quite good; ④Poor. 10). How has your life attitude changed since the pandemic has been under good control? Options: ①More positive; ②Almost the same as before; ③More negative. 11). How often do you spend on focusing on COVID-19 related information? Options: ①Never; ②Only sometimes; ③Often; ④Always. 12). Are you scared or anxious or confused about COVID-19 related news? ①Never; ②Only sometimes; ③Often; ④Always.

**Results**

Most of the students (71.3%) considered that the efficiency of studying at school was better than online-study during home quarantine (Question 1). 59% of them thought that the interactions between students and teachers during school time became more active than online-study during quarantine (Question 4). Nearly 2/3 students had a good relationship with their family and classmates since the pandemic being under good control (Question 8 and 9). More than half of the students got a more positive life attitude after the pandemic (Question 10). Most of the students (68.9%) spent little time focusing on COVID-19 related information (Question 11) and few students (1.9%) often or always felt scared or anxious or confused about COVID-19 related news (Question 12). See **Table S3** for more details.

**Table S1** The rate of different depressive symptoms in high school students assessed by PHQ-9. [*N* (%)]

| Items | Not at all | Several days | More than half  the days | Nearly everyday |
| --- | --- | --- | --- | --- |
| Little interest or pleasure in doing things | 664 (59.9) | 406 (36.6) | 27 (2.4) | 11 (1.0) |
| Feeling down, depressed, or hopeless | 697 (62.6) | 396 (35.7) | 7 (0.6) | 8 (0.7) |
| Trouble falling or staying asleep, or sleeping too much | 827 (74.6) | 251 (22.7) | 22 (2.0) | 8 (0.7) |
| Feeling tired or having little energy | 644 (58.1) | 427 (38.5) | 24 (2.2) | 13 (1.2) |
| Poor appetite or overeating | 789 (71.2) | 289 (26.1) | 23 (2.1) | 7 (0.6) |
| Feeling bad about yourself - or that you are a failure or have let yourself or your family down | 752 (67.9) | 319 (28.8) | 28 (2.5) | 9 (0.8) |
| Trouble concentrating on things, such as reading the newspaper or watching television | 794 (71.7) | 282 (25.5) | 24 (2.2) | 8 (0.7) |
| Moving or speaking so slowly that other people could have noticed. Or the opposite - being so fidgety or restless that you have been moving around a lot more than usual | 919 (82.9) | 170 (15.3) | 10 (0.9) | 9 (0.8) |
| Thoughts that you would be better off dead, or of hurting yourself in some way | 991 (89.4) | 105 (9.5) | 9 (0.8) | 3 (0.3) |

PHQ-9: Patient Health Questionnaire 9-item.

**Table S2** The rate of different anxious symptoms in high school students assessed by GAD-7. [*N* (%)]

| Items | Not at all | Several days | More than half  the days | Nearly everyday |
| --- | --- | --- | --- | --- |
| Feeling nervous, anxious or on edge | 698 (62.2) | 375 (33.8) | 25 (2.3) | 10 (0.9) |
| Not being able to stop or control worrying | 829 (74.8) | 250 (22.6) | 18 (1.6) | 11 (1.0) |
| Worrying too much about different things | 788 (71.1) | 286 (25.8) | 24 (2.2) | 10 (0.9) |
| Trouble relaxing | 795 (71.8) | 278 (25.1) | 22 (2.0) | 13 (1.2) |
| Being so restless that it is hard to sit still | 913 (82.4) | 174 (15.7) | 17 (1.5) | 4 (0.4) |
| Becoming easily annoyed or irritable | 718 (64.8) | 338 (30.5) | 41 (3.7) | 11 (1.0) |
| Feeling afraid as if something awful might happen | 916 (82.7) | 176 (15.9) | 12 (1.1) | 4 (0.4) |

GAD-7: Generalized Anxiety Disorder scale.

**Table S3 The self-evaluation of general recent-status in high school students. [*N* (%)]**

| Items | ① | ② | ③ | ④ |
| --- | --- | --- | --- | --- |
| What do you think of the efficiency of studying at school compared with the online-study during home quarantine? | 790 (71.3) | 212 (19.1) | 106 (9.6) | - |
| How long do you study at school every day? | 351 (31.7) | 522 (47.1) | 205 (18.5) | 30 (2.7) |
| Could you finish your homework on time? | 563 (50.8) | 491 (44.3) | 39 (3.5) | 15 (1.4) |
| How is the interaction between you and your teachers during school study compared with online-study during quarantine? | 654 (59.0) | 352 (31.8) | 75 (6.8) | 27 (2.4) |
| How much could you master from the online-study? | 298 (26.9) | 691 (62.4) | 105 (9.5) | 14 (1.3) |
| Are you tired of studying at school? | 602 (54.3) | 478 (43.1) | 18 (1.6) | 10 (0.9) |
| Compared with studying at school in a normal way, do you like remote online-study better? | 555 (50.1) | 489 (44.1) | 50 (4.5) | 14 (1.3) |
| How is your relationship with your family since the pandemic being under good control? | 690 (62.3) | 349 (31.5) | 63 (5.7) | 6 (0.5) |
| How is your relationship with your classmates since returning to school? | 714 (64.4) | 338 (30.5) | 55 (5.0) | 1 (0.1) |
| How has your life attitude changed since the pandemic has been under good control? | 648 (58.5) | 442 (39.9) | 18 (1.6) | - |
| How often do you spend on focusing on COVID-19 related information? | 248 (22.4) | 514 (46.4) | 281 (25.4) | 65 (5.9) |
| Are you scared or anxious or confused about COVID-19 related news? | 808 (72.9) | 281 (25.4) | 17 (1.5) | 2 (0.2) |
